# Protocol for stereotaxic targeting of the dorsal vagal complex in mice

**DOI:** 10.1016/j.xpro.2026.104807

**Published:** 2026-08-27

**Authors:** Barbara Robar, Holly E. Smith, Lora K. Heisler, Beatrice M. Filippi, Pablo B. Martinez de Morentin

**Affiliations:** 1 School of Biomedical Sciences, Faculty of Biological Sciences, University of Leeds, Leeds, UK; 2 The Rowett Institute, School of Medicine, Medical Sciences and Nutrition, University of Aberdeen, Aberdeen, UK

**Keywords:** Health Sciences, Model Organisms, Neuroscience, Systems biology

## Abstract

The dorsal vagal complex (DVC) is a critical brainstem hub for gut-brain communication, yet detailed accounts for targeting it in mice remain lacking. Here, we present a protocol for bilateral DVC injections via the atlanto-occipital membrane joint. We describe steps for exposing and dissecting the atlanto-occipital membrane, bilateral administration of injectable agents into the nucleus of the solitary tract (NTS), closure of the wound, and post-operative monitoring. This protocol enables studies using tracings, optogenetics, chemogenetics, and related neuroscience techniques.

## Highlights

• First step-by-step protocol for stereotaxic access to the mouse dorsal vagal complex
• Obex-referenced coordinates guide bilateral injection into this brainstem region
• Viral reporter validation confirms accurate and reproducible targeting

## Abstract (editor highlights)

Publisher’s note: Undertaking any experimental protocol requires adherence to local institutional guidelines for laboratory safety and ethics.

## Graphical abstract

**Figure:**
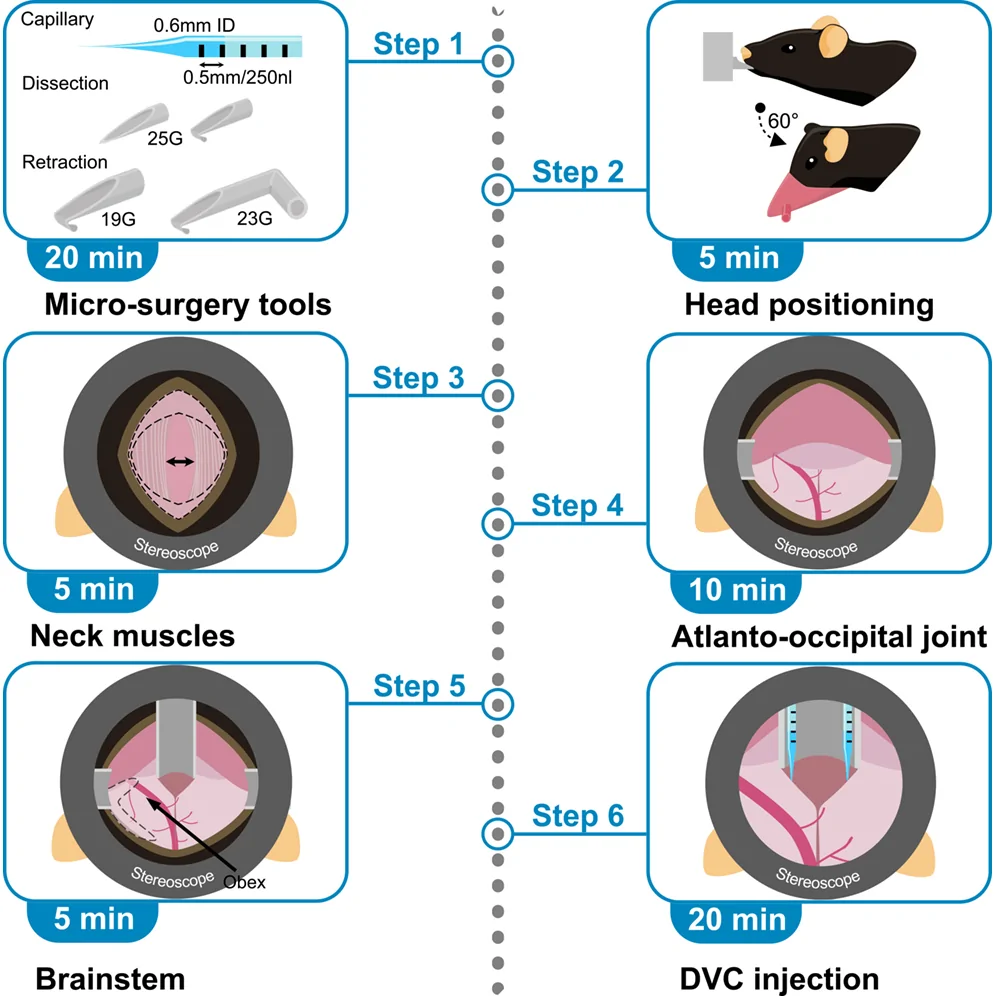

## Before you begin

The Dorsal Vagal Complex (DVC) is the primary central relay for vagal afferent information from the gastrointestinal tract, cardiovascular system, and respiratory organs. It is comprised of the nucleus of the solitary tract (NTS), the area postrema (AP) and the dorsal motor nucleus of the vagus (DMX). Subpopulations of the DVC neurons regulate feeding behavior, blood pressure, respiration, and metabolic homeostasis. Recent studies have identified numerous cell-type-specific populations within the DVC through their direct manipulation including, Bdnf,^1^ Ppg,^2^ Glp-1r,^3^^,^^4^ Calcr,^5^^,^^6^ Cck,^7^ ChaT,^8^ Th,^9^ A2,^9^^,^^10^ NE,^11^ Lepr,^12^ InsR,^13^AgRP,^14^ ^16^Pomc,^15^ GIPR,^16^ Gfral,^17^^,^^18^ Adcyap1,^19^^,^^20^ 5-ht2cr,^21^ Prlh^22^ and astrocytes.^23^

We have recently demonstrated that GABAergic neurons in the DVC (GABA^DVC^) project to the hypothalamic arcuate nucleus (ARC) and regulate food intake and body weight via direct inhibition of NPY/AgRP neurons.^24^ We followed a stereotaxic surgical intervention to reach the DVC that enabled several circuit-based interrogations to define the role of this neuronal subpopulation in feeding behavior and body weight. Specifically, we manipulated Vgat^DVC^ cells using chemogenetic activation and silencing (hM3Dq and hM4Di), visualized Vgat^DVC^ axonal projections using channel rhodopsin assisted circuit mapping (CRACM) and performed specific manipulation of Vgat^DVC-ARC^ axonal terminals using opsins (ChR2).

### Innovation

**Figure 1:**
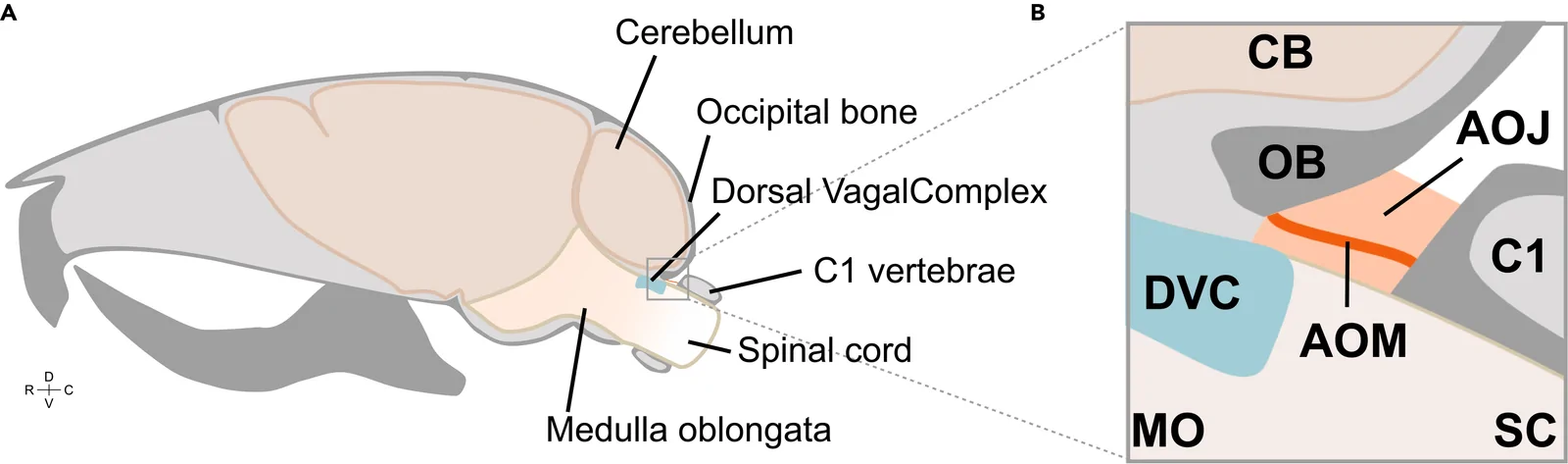
Location of the atlanto-occipital membrane (A) Sagittal view of a mouse skull and brain depicting the cerebellum, occipital bone, cervical vertebrae C1, medulla oblongata and dorsal vagal complex. (B) Representation of the atlanto-occipital membrane spanning the atlanto-occipital joint between the base of the occipital bone and the anterior part of the C1. AOJ, Atlanto-Occipital joint; AOM, Atlanto-Occipital membrane; CB, Cerebellum; C1, Cervical vertebrae; DVC, Dorsal Vagal Complex; MO, medulla oblongata; OB, Occipital bone; SC, spinal cord.

The stereotaxic delivery of viral vectors into the DVC of mice is technically challenging since it is positioned below the cerebellum (Figure 1) and follows a fundamentally different protocol from standard stereotaxic injections. First, the DVC is located on the dorsal surface of the brainstem, requiring access through the posterior fossa rather than through the skull vault. This necessitates flexion of the head and dissection of the atlanto-occipital membrane. Second, the NTS is a small, elongated nucleus flanked by the AP dorsally and the DMX ventrally, creating a narrow corridor for accurate targeting where off-target delivery has significant functional consequences. Third, unlike bregma-referenced coordinates used for brain targets, this current protocol requires the obex (*calamus scriptorius*) as the stereotaxic zero-point, a surface landmark that must be visually identified under magnification after membrane dissection. In our hands, this obex-referenced approach reproducibly localizes the injected agent to the medial NTS. Although, there are reports using a the atlanto-occipital approach,^25^ they only provide details to target ventral brainstem regions such as the hypoglossal nucleus or the glomerular reticular nucleus. Given the increasing interest of accessing the DVC to study gut–brain signaling, sympathetic control, and energy homeostasis, we provide a validated step-by-step protocol that allows targeting this brainstem region.

### Institutional permissions

Animal handling and experimentation was carried out according to UK Home Office guidelines and the requirements of the United Kingdom Animals (Scientific Procedures) Act 1986 and the University of Leeds animal welfare ethical review board. Mice were housed under a 12:12 h light/dark cycle with free access to food and water. Two 12-weeks-old *Vgat*^*Cre*^ (JAX016962) male mice were used for this protocol.

***Note:*** Before applying this protocol in live animals, appropriate ethical approval must be obtained from relevant institutional and government authorities.

### Preparation of administering agent

The selection of the administering agent will depend on the stage of the experimental approach. For initial steps to acquire the necessary skills to perform this procedure we advise using validation agents. If by contrast, the researchers is competent for this protocol and wants to proceed with the experimental approach, a careful selection of the neuronal tracing and manipulation tools needs to be chosen.

***Note:* Validation agents.** We recommend the use of cresyl violet solution for target validation on post-mortem animals. Cresyl violet allows rapid visualization of targeted region accuracy using a brightfield microscope and enables the visualization of the administered agent spread using 550nm filter in fluorescence microscope. Alternative to cresyl violet, other dyes commonly available in laboratories suitable for this validation include trypan blue and methylene blue (both with brightfield and fluorescence visualization using red or far-red filters) and India Ink with only brightfield visualization.

***Note:* Experimental agents** (e.g., viral particles). A viral titration and expression study must be performed to define the optimal conditions.^26^^,^^27^^,^^28^^,^^29^

### Preparation of glass capillary

**Timing: 20 min**

This step describes how to prepare the injection method using pulled glass capillaries.

1. Pull glass capillaries using a micropipette puller to achieve a tip diameter of approximately 40 μm.

***Note:*** Using Narishige GD1 capillary on a Sutter Instruments P-97 puller an ideal pulling program would be: Heat=600, Filament=4, Velocity=60, Delay=145, Pull=175.

2. Inspect the tip under a microscope to verify diameter and confirm the tip is not broken or jagged.

***Note:*** Whether using an automatic or manual injector, we advise marking the capillary with graded lines of volumes.

***Note:*** If using G1 capillary (OD=1mm, ID=0.6mm), a 1mm mark interval corresponds to approximately 250nl.

3. Fill the capillary with the administering agent by gentle aspiration using an microinjector (e.g., pneumatic IM-11-2 Narishige).

**CRITICAL:.** Before loading the agent, review the prefilling requirements of the injector used. Oil microinjectors require prefilling the capillary with specific oil, whereas pneumatic microinjectors do not.

4. Test-eject a small volume to confirm flow before mounting on the stereotaxic arm.

**CRITICAL:.** We strongly advise performing test-eject practice with solutions of comparable viscosity to the solution to be used in the experimental approach.

### Preparation of the surgery setup

**Timing: 30 min**

This step describes the preparation of the surgical tools and instruments and sterile conditions.

**CRITICAL:.** Before performing this protocol on an experimental animal, successful targeting must be confirmed using validation agents (see above) in animals post-mortem.

***Note:*** We recommend practicing the sterile conditions even during post-mortem training.

**Figure 2:**
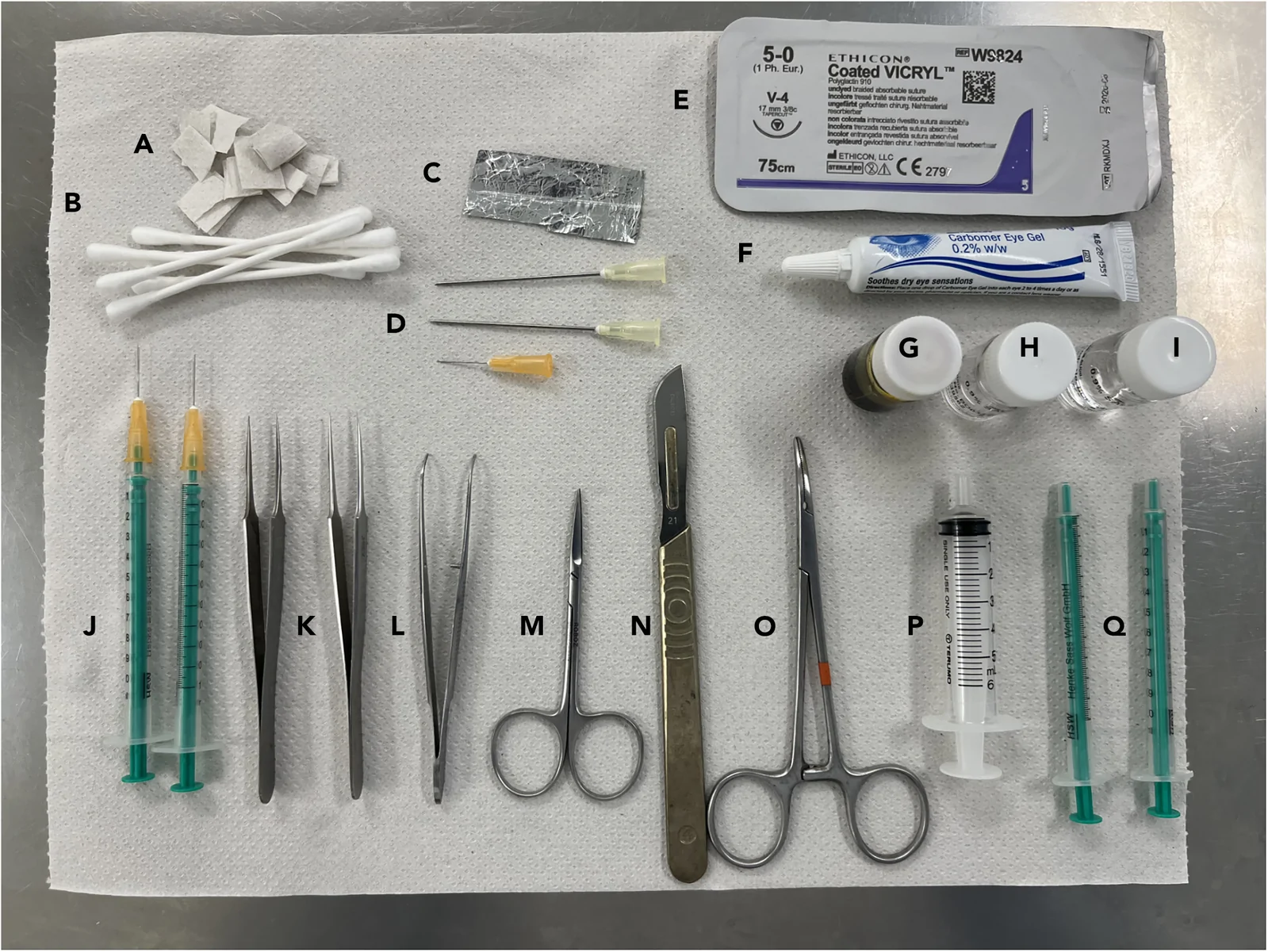
Surgical tools (A–Q) Absorbent paper, (B) Cotton buds, (C) Graded capillary, (D) retractor-needles, (E) Absorbent suture, (F) Eye lubricant, (G) Iodine Povidone, (H) Saline, (I) Analgesic, (J) Dissecting needles (scalpel and hook), (K) Fine dissecting forceps, (L) Fine dissecting forceps with teeth, (M) Fine dissecting scissors, (N) Scalpel, (O) Fine needle holder, (P) 5 mL syringe, (Q) 1 mL syringes.

**Figure 3:**
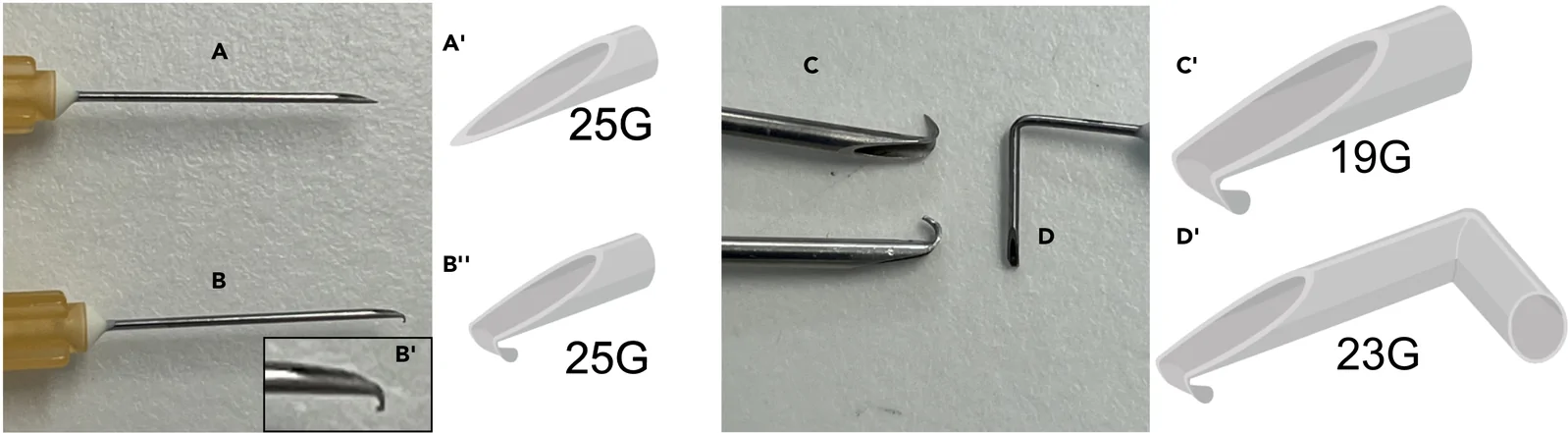
Modified dissecting needles and diagrams (A) scalpel, (B) hook, (C) muscle retractors and (D) cerebellum retractor.

5. Surgery sets.
  a. A set of standard surgical tools and fluids (Figure 2) including:
    i. Absorbent paper.
    ii. Cotton swab sticks.
    iii. Graded capillary.
    iv. Retractor needles.
    v. Absorbent suture (5-0).
    vi. Eye lubricant (0.2% carbomer).
    vii. Iodine povidone, 7.5%.
    viii. Saline (0.9%).
    ix. Analgesic.
    x. Dissecting needles.
    xi. Fine dissecting forceps.
    xii. Fine dissecting forceps with teeth.
    xiii. Fine dissecting scissors.
    xiv. Scalpel.
    xv. Fine needle holder forceps.
    xvi. 5ml syringe.
    xvii. 1m syringe.
  b. A set of custom dissecting needles (Figure 3) modified to be used as:
    i. Scalpel.
    ii. Hook.
    iii. Muscle retractor.
    iv. Cerebellum retractor.
  c. Surgical gowns and drapes.
  d. Equipment.
    i. Stereotaxic frame with syringe holder.
    ii. Heat pad with thermostat.

***Note:*** Use transparent self-seal sterilization pouches for each reusable set.

***Note:*** If performing multiple surgeries in one day, prepare two complete surgical sets and use a glass bead sterilizer in between surgeries. This allows sterilizing one set while performing surgery with the other set.

**Figure 4:**
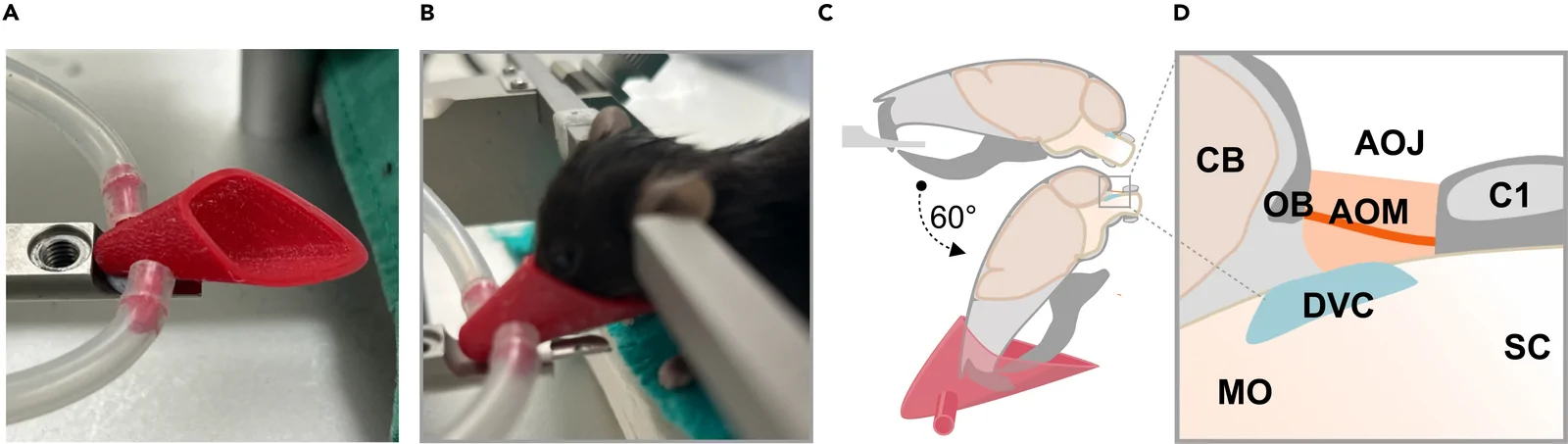
Angled head with custom anesthetic mask (A) 3D printed anesthetic mask. (B) Representative position of the mouse head angled. (C) Diagram of the head angled 60°. (D) Diagram of the atlanto-occipital joint with the head angled. AOJ, Atlanto-Occipital joint; AOM, Atlanto-Occipital membrane; CB, Cerebellum; C1, cervical vertebrae 1; DVC, Dorsal Vagal Complex; MO, medulla oblongata; SC, spinal cord.

6. Animal preparation.
  a. Include animal details in the surgery record’s book.
  b. Set the recovery heating cabinet at approximately 35°C and verify with an independent thermometer.
    ***Note:*** Placing a temperature-controlled heating mat (37°C) underneath the recovery cage is an effective method to provide a warm environment during the recovery period while the mouse regains normal awake behavior.
  c. Prepare new home cages with standard enrichment. Mash standard pelleted diet in warm water (35°C) and place in a petri dish inside the new home cage to provide palatable, easy to eat food.
7. Surgical station.
  a. Check all surgical tools are autoclaved, dried, and cool.
    ***Note:*** Given the unusual positioning of the mouse head (60° forward) rodent anesthetic masks and tooth restrainers on most stereotaxic frames are not suitable for DVC surgery. We have 3D printed a mask that accommodates the variable angle of the head (Figure 4A). Similar anesthetic masks can be obtained from specialist suppliers.
  b. Confirm adequate anesthetic and oxygen supply and retrieval thought the mask.
  c. Pre-fill a 1 mL syringe with warm (35°C) sterile 0.9% saline; place on a heat mat (35°C) (for surgical subcutaneous rehydration).
  d. Pre-fill a 5 mL syringe with sterile saline; place in a beaker on wet ice for bleeding control and wound cleaning.
  e. Clean the stereotaxic frame and surgical area with 70% ethanol.
  f. Place sterile surgical drapes.
8. Surgeon gowning and preparation.

Follow standard aseptic gowning procedure and principles (e.g., LASA, RAT), as described elsewhere.^30^

### Pre-operative care and mouse positioning

This step describes the preparation of the mouse using inhaled anesthesia.

***Note:*** Inhaled anesthesia offers many benefits over injectable anesthesia, including control of anesthetic depth and reversible state and a faster recovery time for the animal. Moreover, injectable anesthesia is invasive and displays strain and sex variability, making it more unpredictable with higher mortality rates. For these reasons, we advise using inhaled over injectable anesthesia, but we acknowledge that ethical approvals and specialized equipment may be required.

***Alternatives:*** This protocol is fully compatible with injectable anesthesia.

9. Induction of anesthesia.
  a. Click or tap here to enter text. Click or tap here to enter text.Place the mouse in the pre-anesthetic chamber and set the anesthetic flow (3-5% isoflurane in oxygen).
  b. Once fully anesthetized as illustrated by the lack of pedal withdrawal reflex, transfer the mouse to the stereotaxic frame and place the nose in anesthetic the mask.
10. Maintenance of anesthesia and pre-operative preparation.
  a. Maintain isoflurane at 1%–2% in oxygen throughout the procedure.
  b. Secure head to the stereotaxic frame using ear bars.
  c. Clean fur and disinfect with cotton wool with povidone-iodine.
    ***Note:*** We advise a thorough cleaning and disinfection and use electric fur clipping as hair removal as the preferred method over shaving with a razor blade as the latter creates micro-abrasions associated with a higher risk of infections.
  d. Apply eye hydration (e.g., carbomer, directly to the eyeball) and provide light protection.
  e. Apply analgesic and anti-inflammatories.
    **CRITICAL:** The preferred pharmacological regime must be approved by the named veterinarian or relevant body at the institution. The DVC, specifically the DMX, is involved in lowering heart^31^ and breathing rate^32^^,^^33^^,^^34^ and termperature^35^ in certain stress situations that induce tachycardia and hyperventilation. Once animal’s vitals are steady and the surgical procedure begins, we have not observed changes in breathing or heart rate associated with the direct manipulation of the DVC during the surgical procedure. Nevertheless, we strongly advise considering the expected outcomes of the experimental manipulation with a balanced pharmacological approach to prevent further rate reduction specially affecting the early recovery period.
  f. Apply pre-warmed (35°C) hydration fluid saline (1 mL/30 g mouse, SC).
11. Positioning of the head.

***Note:*** At this step, the DVC protocol diverges from a standard stereotaxic brain surgery protocol. From a position where the skull is horizontal, incline the head forward approximately 60 degrees (Figures 4B–4D). Adjust the mask to the new position and make sure it is sealed on the mouse’s snout.

**CRITICAL:** The head flexion angle is critical. If the angle is low, the atlanto-occipital membrane will not be accessible. If too high, the mouse’s airway may become obstructed. troubleshooting 1.

***Note:*** Once the head is flexed, if a click is heard with each of the mouse’s inhalation, reduce the angle immediately. This noise signifies that the trachea is being squeezed by the head angle. It is essential to monitor breathing continuously.

## Key resources table

**Table:** 

REAGENT or RESOURCE	SOURCE	IDENTIFIER
**Bacterial and virus strains**		

AAV-hSyn-DIO-EGFP	Addgene	Addgene AAV9-50457
AAV-hSyn-DIO-mCherry	Addgene	Addgene AAV9-50459

**Chemicals, peptides, and recombinant proteins**		

Isoflurane	MERCK	792632
Carprofen	Rimadyl™, Zoetis	N/A
Sterile 0.9% sodium saline	Cambridge Bioscience	TSH-00526
Iodine-povidone solution	Vetaseot, MedicalXpress	XHG012
Eye lubricant, carbomer eye gel 0.2%	Medicom	--
Cresyl violet dye	MERCK	C5042
Ethanol	Fisher Scientific	Cas# 64-17-5
Virucidal disinfectant	Virkon™	12338667
Sterile Surgical Adhesive	Skinbond, Animus	AG0102
Sudan Black B	Fisher Scientific	DRE-C16986110

**Experimental models: Organisms/strains**		

12 weeks old VgatCre male	Centre of Biomedical Services	IMSR_JAX:016962

**Software and algorithms**		

Fiji	ImageJ	https://imagej.net
AffinityDesigner	Affinity	https://www.affinity.studio/

**Other**		

Fine dissecting scissor	MERCK	S3146
Fine dissecting forcepts	MERCK	F4017
Needle Holder	WPI	14110
Absorbent paper	Whatman	1440329
Cotton buds	Camlab Limited	1234602
Stereotaxic Instrument (Kopf 942)	Kopf	900SL
Pneumatic Microinjector	Narishige	IM-11-2
Glass Capillaries	Narishige	GD-1
Pipette puller	Sutter Instruments	P-97
Micro syringe	Hamilton Bonaduz	7632-01
Removable needles 34G	Hamilton Bonaduz	207434
Suture 6/0 absorbable	Ethicon	ETHW9913
Needle 19G	Greiner Bio-One	N1950
Needle 25G	Greiner Bio-One	N2516
Needle 23G	Greiner Bio-One	N2316
Syringe 1ml	SLS	SYR6200
Syringe 5ml	SLS	SYR6204
Stereo microscope	Zeiss	OPMI pico
Sterilisation Pouches	Fisher Scientific	NWKP0915
Heating pad with thermostat	Amazon	N/A

## Step-by-step method details

### Exposure of the atlanto-occipital joint

**Timing: 10–15 min**

This section describes the surgical approach to expose the dorsal brainstem surface through the atlanto occipital joint. This approach is fundamentally different from a standard stereotaxic protocol and requires practice with cadaver tissue before undertaking on live animals. The entire process is performed using a stereo microscope.

**Figure 5:**
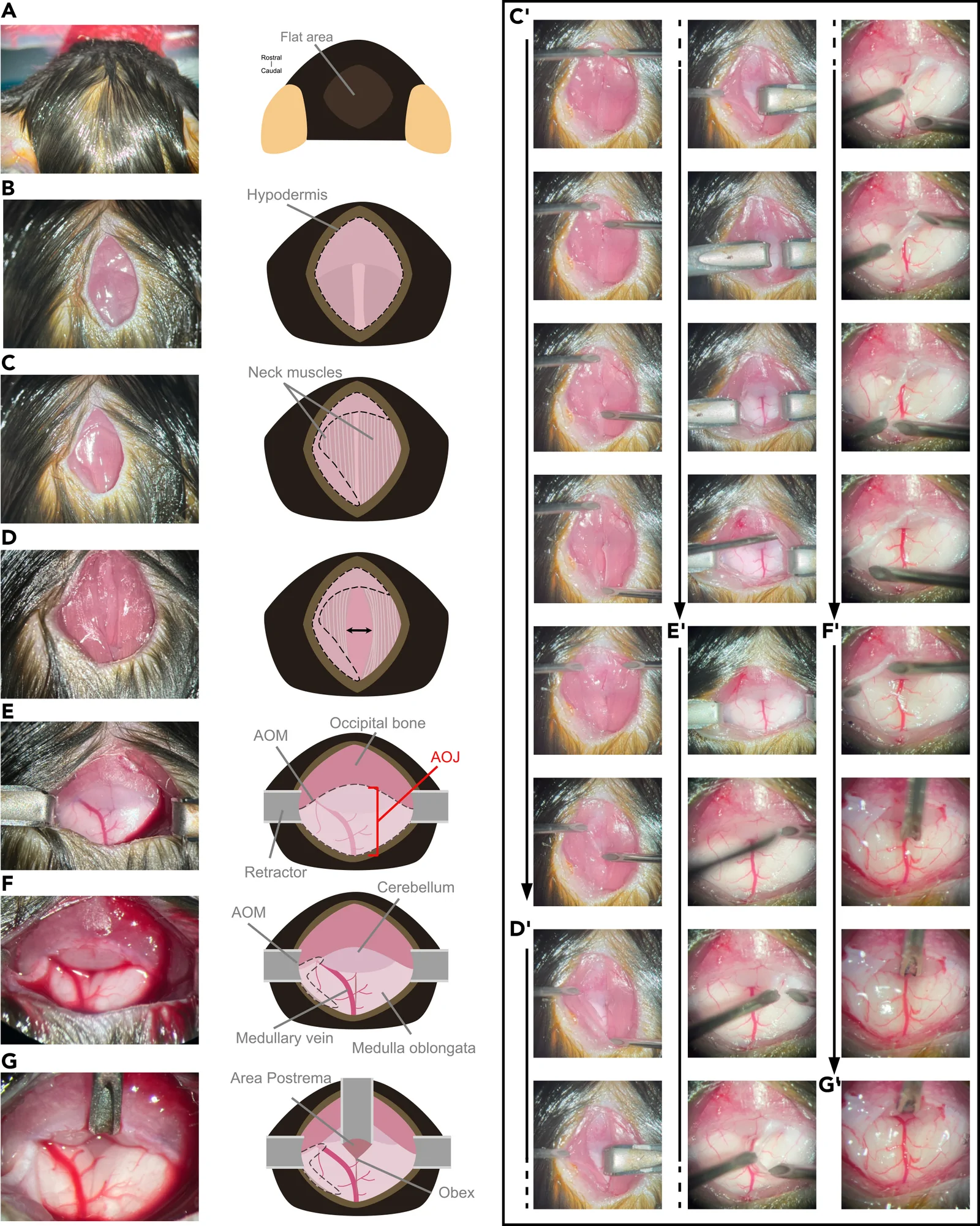
Dissection of the atlanto-occipital membrane and access to the dorsal branstem (A) Identification of virtual flat at the back of the neck. (B) Incision on the skin and exposure of hypodermis. (C) Dissection of hypodermis and exposure of neck muscles. (D) Separation of the muscles. (E) identification of AOM and AOJ. (F) Dissection of AOM. (G) Retraction of cerebellum. (C′ to G′) Panel with detailed dissection images taken from video captures in Methods Videos S1, S2, S3, S4, S5, and S6.

***Note:*** This flat region is due to the distension of the neck stretching the skin between the skull bone and the C1 vertebrae.

1. Identify a flat horizontal area on the surface of the skin (Figure 5A).

2. Pinch the skin with the forceps, lift the skin and cut it longitudinally with the pair of surgical scissors to create a 0.5cm incision (Figures 5B and Methods Video S1. Incision).

***Note:*** This will reveal the hypodermis.

Methods Video S1. Incision, relevant to Exposure of the dorsal brainstem, Step 1b

Methods Video S2. Hypodermis, relevant to Exposure of the dorsal brainstem, Step 1c

Methods Video S3. AO Joint, relevant to Exposure of the dorsal brainstem, Step 1f

**Tip:** We will use retractors, so as little as 0.5 cm would be sufficient for a skilled surgeon and will accelerate the wound recovery.

***Optional:*** If the experimental approach involves accessing a second brain structure during the same surgery session, (e.g., transduction of viral particles into the DVC and placement of an optic fiber above the arcuate nucleus of the hypothalamus^24^) we advise a larger incision (1 cm). This will allow accessing a second brain structure using the same skin opening with the benefit of reduced risk of infection and improved recovery times.

3. Cut the hypodermis following a process as detailed in section b (Figures 5C and Methods Video S2).

***Note:*** This will reveal the right and left neck muscles (semispinalis capitis) running from the base of the occipital bone to the cervical vertebrae C1. A white pale midline is visible between the muscles

4. Using the *scalpel* and the *hook needles* cut along the midline and gently pull the left and right muscle apart (Figures 5C’ and 5D’).
5. Using the *scalpel needle*, detach the ventral layer of the neck muscles from the membrane and pull the muscle further apart using the retractor. Repeat this process for the other neck muscle (Figures 5D’ and 5E’).
6. Secure the separated muscles using *muscle retractors* (Figures 5E and Methods Video S3).
  ***Note:*** This will allow you to visualize the posterior atlanto-occipital membrane (AOM), which is a translucent layer spanning the base of the occipital bone and the C1, protecting the atlanto-occipital joint (AOJ)
  **CRITICAL:** Given the limited anatomical space, we advise using needles (19G) with their tips bent down as retractors. Their tips must be also blunted to prevent damaging the muscle or tearing the AOM.
  ***Note:*** At the end of this stage of the procedure, you should have a 1 cm plane to identify:
  a. Top: Occipital bone (pink).
  b. Middle: ventral cerebellum (pale pink).
  c. Bottom: medulla oblongata (white).
  d. Median posterior medullary vein (red).

### Dissecting the atlanto-occipital membrane

**Timing: 10–15 min**

This section describes how to dissect the AOM to reveal access to the AOJ.

***Note:*** The median posterior medullary vein accesses the skull cavity laterally. The dissection must take place on the opposite side of the vein to prevent any damage to the vein.

7. Using the *scalpel needle*, perform a vertical perforation of the AOM at the joint with the occipital bone.

***Note:*** This will relief the inner pressure of the AOJ and release CSF.

**CRITICAL:** The release of intracranial pressure can potentially lead to discomfort and pain during the recovery perido. We strongly advise following a proactive post-operative pain management (Step 32)

8. Using the *hook needle* and a sharp needle as scalpel, dissect the AOM caudally and laterally towards the opposite side (Figures 5E’ and 5F′ and Methods Video S4).

**CRITICAL:** When dissecting close to the vein pull the AOM up using the retractor needle to allow sufficient space for the scalpel to cut the AOM over the vein.

***Note:*** If necessary, gently clean CSF or blood from the area using sterile cotton buds.

***Note:*** A small degree of bleeding is acceptable as a tiny vessel can be damaged during this part of the procedure. However, if the medullary vein is lesioned, as indicated by bleeding that does not stop, then the mouse may suffer a brain stroke during recovery, we advise to immediately humanely kill the mouse. troubleshooting 2.

***Note:*** Once the AOM is completely dissected and the inner pressure is relieved, the medulla and the cerebellum will slightly protrude (Figure 5F).

9. Using the *cerebellum retractor needle*, pull the base of the cerebellum rostrally until the obex is visible (Figures 5F’ to 5G′ and Methods Video S5). troubleshooting 3.

***Note:*** The obex (calamus scriptorius) appears as a small V-shaped notch at the caudal end of the visible brainstem surface. The area postrema is visible as a slightly raised, vascularized region immediately rostral to the obex. The aim is to reveal an area of approximately 2mm between the obex and the retractor (Figure 5G).

10. Secure the retractor at the desired position.

***Note:*** Adequate illumination and magnification are essential. If the surface is obscured by blood or CSF, gently irrigate with saline and absorb with absorbent paper.

Methods Video S4. AO Membrane, relevant to Exposure of the dorsal brainstem, Step 2b

Methods Video S5. DVC, relevant to Exposure of the dorsal brainstem, Step 2c

### Bilateral administration of injectable agents into the NTS

**Timing: 10–20 min**

This section describes how to administer an injectable agent into the dorsal brainstem targeting the medial NTS. The AP and the DMX can also be targeted using the same techniques described here but coordinates for either regions will need to be acquired.

11. Affix the graded glass capillary to the stereotaxic syringe adapter.
12. Load the injectable agent. troubleshooting 4.
13. Load a small volume of air sufficient to prevent the withdrawal of the agent by capillarity when the capillary enters in contact with the CSF.
14. Position the tip of the capillary above but touching the obex (Figure 6A).

**Figure 6:**
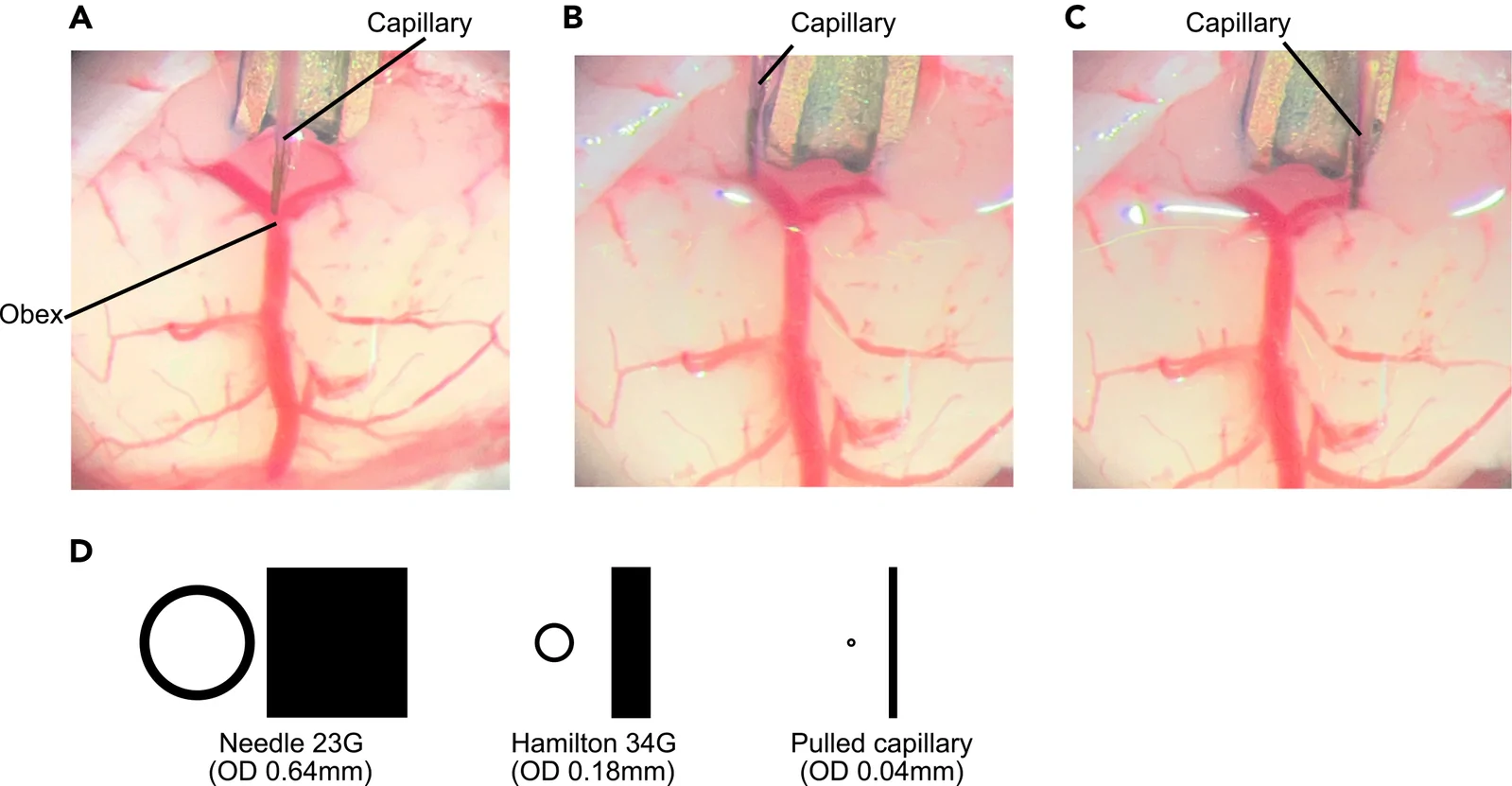
Bilateral administration of injectable agent into the NTS (A) Zero of stereotaxic corrdinates placing the tip of the capillary above the obex; (B) injection on the left side (L:-0.25 mm, DV:-0.25 mm) and (C) injection on the rigth side (L:+0.25 mm, DV:-0.25 mm). (D) Comparison of diameter and cross section between 23G cerebellum retractor needle, Hamilton 34G and capillary.
15. Zero the stereotaxic coordinates to this position.
16. For targeting the NTS: Lift the capillary slightly and move it to the coordinates: antero-posterior -0.25mm and lateral +0.25mm, to the obex. This should leave the tip of the capillary above the commissure between the AP and the NTS (Figure 6B).
17. Displace the capillary ventrally −0.25 mm.
18. Start the administration of 250 nl of agent at rate of 50 nl/min.
19. Leave the capillary on the target position 5 min.
20. Slowly retract the capillary.
21. Move the capillary laterally to the opposite side −0.25 and repeat the injection (Figures 6C and Methods Video S6).

***Optional:*** Microliter syringes can be used, but we recommend using glass capillary pipettes. We provide a cross-section comparison of the thinnest available needle (Hamilton, 34G) and a pulled capillary (GD-1) (Figure 6D).

**CRITICAL:** In some mice, the medullary vein will travel into the brain following the commissure of either side. The vein can be gently pushed to a side with the tip of the capillary. troubleshooting 5.

Methods Video S6. Injection, relevant to Bilateral administration of injectable agents into the NTS, Step 13

### Closure of the wound

**Timing: 10 min procedure**

This section describes how to reposition the semispinalis capitis muscles and suture the skin.

***Optional:*** When performing in live animals a post-surgical monitoring time is required.

22. Clean the AOJ by gently flushing with sterile saline and remove any clotted blood.
23. Slowly reposition the head of the mouse to an almost horizontal plane.
24. Hydrate the neck muscles by flushing saline and perform a gentle massage with aseptic techniques to return them to position.

***Note:*** A small drop of surgical glue can aid in re-bonding both muscles together.

25. Pull the skin together and clean it from inside to outside with a cotton bud with sterile saline.
26. Add stitches of absorbable suture to close the wound.

**CRITICAL:** Animal monitoring is essential during the following 48h to prevent wound opening. troubleshooting 6. Individual housing may be used as a remedy to prevent grooming and wound opening. We acknowledge that this will require ethical approval but also may impact the experimental needs. troubleshooting 7.

### Post-operative monitoring

**Timing: 48 h to 1 week**

***Note:*** Given the DVC’s role in respiratory control, energy balance and gastrointestinal function, animals require closer post-operative monitoring than most other brain targets. We recommend monitoring three aspects for at least 7 days after surgery.

27. Assess welfare using the Mouse Grimace Scale at least once daily for the first week, alongside condition indicators including posture, coat, activity and respiratory pattern.

**CRITICAL:** Continue the approved analgesic and anti-inflammatory regimen. Because this protocol involves the release of CSF during the membrane dissection and potential reduction of the intracranial pressure, we advise a proactive rather than reactive analgesic approach.

**CRITICAL:** Brainstem surgery carries risks of respiratory depression and swallowing dysfunction. If weight loss exceeds approved threshold or respiratory distress is observed, apply humane endpoint criteria immediately. troubleshooting 8.

28. Record body weight before surgery and daily throughout recovery.

**CRITICAL:** Weight loss is expected due to the anesthesia and the surgical intervention; however, further weight reduction (or weight gain) can be anticipated when targeting the DVC as an expected scientific outcome. If unexpected, apply humane endpoint criteria if weight loss exceeds the approved threshold.

29. Monitor food intake and hydration during recovery.

**CRITICAL:** Reduction in eating is expected during the first 48 h after surgery, but changes in eating patterns can be anticipated when targeting the DVC as an intended experimental outcome. If changes in food intake are unexpected, apply humane endpoint criteria if weight loss/gain exceeds the approved threshold.

## Expected outcomes

Before performing experimental surgeries, extensively validate coordinates and technique by injecting cresyl violet dye into a mouse cadaver. Successful targeting is confirmed when dye is localized to the medial NTS with minimal spread into the AP or DMX. troubleshooting 9. Nonetheless, the ultimate validation of the current protocol involves confirmation of agent expression, spread and readout of the expected experimental outcome.

**Figure 7:**
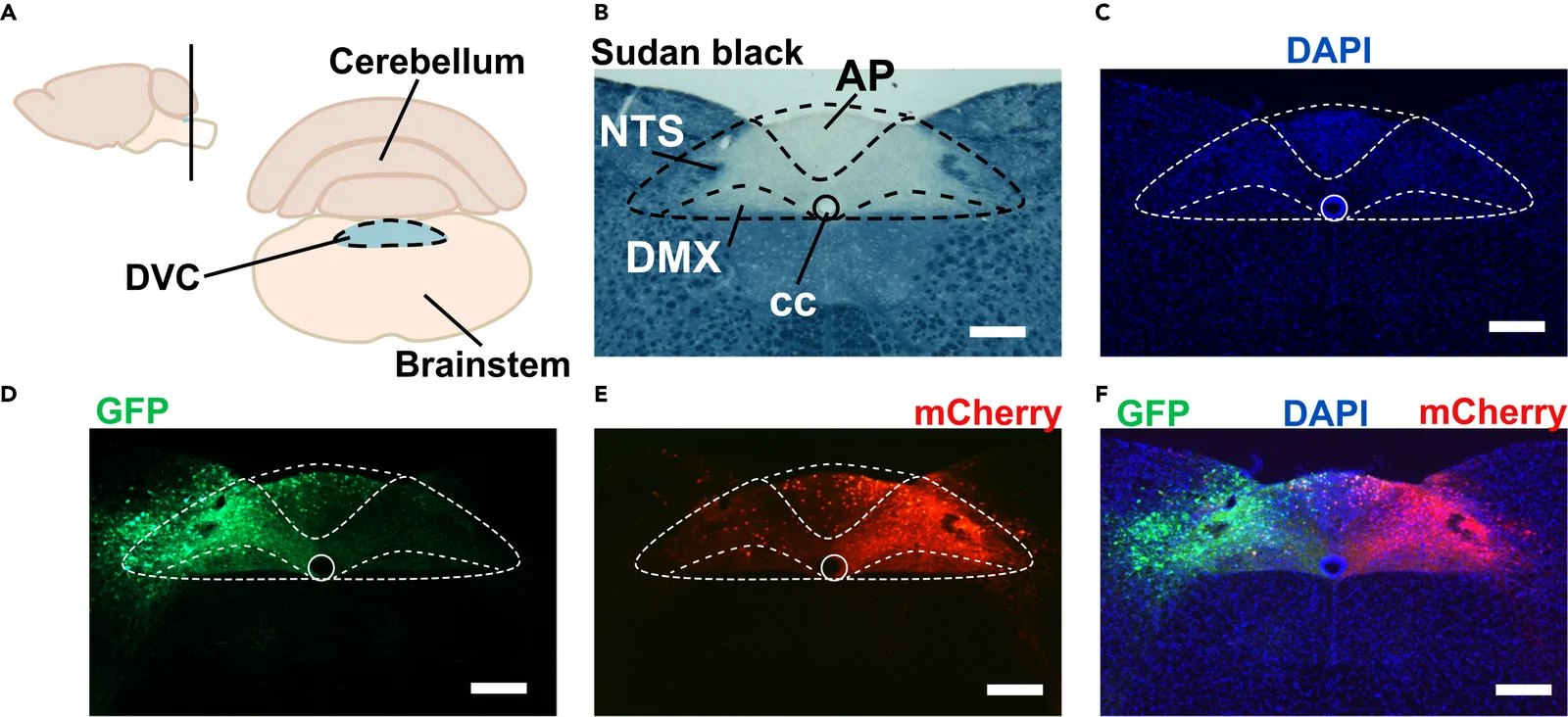
Representative DVC targeting with AAV expressing reporters (A) Diagram of coronal section of the brainstem containing the DVC. (B and C) Representative photomicrographs of a coronal section of a *Vgat*^*Cre*^ mouse transduced with AAV-hSyn-DIO-EGFP on the left side and AAV-hSyn-DIO-mCherry on the right side of the DVC, (B) counterstained with Sudan black B, (C) counterstained with DAPI, (D) GFP signal, (E) mCherry signal and (F) merged. Scale bar 200 μm. AP, area postrema; cc, central canal; NTS, Nucleus of the Solitary Tract; DMX, Dorsal Motor Nucleus of the Vagus.

For experimental animals, successful viral transduction is indicated by bilateral reporter expression restricted to the NTS, spanning 4–6 coronal sections (∼200–400μm). We have transduced a *Vgat*^*Cre*^ mouse with viral particles into the right NTS expressing red (AAV-hSyn-DIO-mCherry) (Figure 7A) and into the left NTS expressing green (AAV-hSyn-DIO-GFP) (Figure 7B) reporters. Eight weeks after viral transduction, mouse was perfused, brain was dissected and coronally sectioned and sections were imaged under the fluorescence microscope (Figure 7).

The accuracy of DVC injection has been further validated by functional readouts published.^24^

## Limitations

We acknowledge that there exist several limitations of technical nature when performing this surgical procedure.

The primary limitation is the surgeon’s skills. A critical step comprises the dissection of the AOM without damaging the medullary vein. This can otherwise lead to the need to humanely kill the animal. It is essential to practice technique competency in cadavers before moving to live animals.

The DVC is an elongated structure, and it is formed by three main structures: the AP, the NTS and the DMX. If your study design uses an intersectional approach (e.g., Cre/LoxP technology) we recommend carefully reviewing the off-target possibilities. We highly recommend optimizing: 1) agent titer/concentration, 2) the volume of the agent administered and 3) adapting the coordinates from the obex.

The transduction volume is constrained to small amounts. Exceeding 300nl risks off-target transduction. For broader coverage, multiple injection sites along the anterior-posterior axis P axis may be considered.

## Troubleshooting

### Problem 1

Mouse with dyspnea and/or gasping (clicking noise).

### Potential solution

• Reduce the concentration of anesthetic.
• Lower the angle of the head.
• Prevention: verify consciousness and angle the head slowly.

### Problem 2

Excessive bleeding during membrane dissection.

### Potential solution

• Apply cold sterile saline; wait 1–3 min. Apply gentle pressure with absorption paper for 3-5 min. If the bleeding is from the medial posterior medullary vein, consult the veterinary or the named animal welfare officer.
• Prevention: use only blunt dissection needles, avoid lateral excursions, ensure adequate visualization.

### Problem 3

Inability to identify the obex.

### Potential solution

• Irrigate with room-temperature sterile saline and absorb with sterile cotton buds.
• Improve illumination and magnification.
• Verify head angle (∼60°).
• Use a deeper retractor for the cerebellum.
• Lower the thorax.
• If the obex cannot be identified, do not proceed and humanely kill the animal.

### Problem 4

Blocked capillary.

### Potential solution

Given how superficial the target region is, the tip of the capillary can be cut several times if it is blocked while maintaining its diameter.

### Problem 5

Medullary vein above the commissure AP-NTS.

### Potential solution

• Use a blunt fine tool to displace it during the positioning of the capillary.
• Use the capillary to push the vein to the side.

### Problem 6

Wound re-opening.

### Potential solution

• When suturing, place the first knot close to skin with two additional knots.
• Add a drop of surgical glue to the knot.
• Re-suture once if stitches are removed within the first 24 h. If beyond 24 h or infected, apply humane endpoints. This may be more recurrent in female mice due to social grooming behavior. One option is to single house female mice for 48h until wound has healed.

### Problem 7

Undesired Single housing.

In some situations, the researcher needs to isolate the animals to prevent social grooming that can open the wound (Problem 6), damaging the brain implants (e.g., for optogenetic, photometry or drug delivery) or for male fighting. However, single housing is not recommended as animals could develop stereotypical behaviors that can alter or confound the expected outcomes and increase metabolic and cognitive variability. Moreover, the period of single housing may need to be approved by the ethical committee.

### Potential solution

Providing an enriched environment including nesting material, a tunnel per mouse, and foraging toys can prevent the need of single housing. Moreover, this approach has enabled sequences of single and group housing experimental conditions in both male and female mice.

### Problem 8

Post-operative respiratory distress or mortality.

### Potential solution

• This may indicate brainstem trauma. Ensure DV injection is conservative (−0.25 mm max).
• Do not exceed 300nl of agent volume and at 50nl/min of infusion rate.
• Maintain mouse body normal temperature and hydration.
• We strongly recommend the use of pulled glass capillaries over specialized commercial steel needles. The thinnest 34G stainless steel needles available are still 4x wider that pulled capillaries.

### Problem 9

Off-target expression of the agent in the brain.

### Potential solution

• Reduce volume of agent.
• Reduce the administration rate.
• Increase micropipette insertion time to 10 min.
• Re-adjust the coordinates.

## Resource availability

### Lead contact

Further information and requests should be directed to Pablo B. Martinez de Morentin (p.demorentin@leeds.ac.uk).

### Technical contact

Technical questions should be directed to Pablo B. Martinez de Morentin (p.demorentin@leeds.ac.uk).

### Materials availability

This protocol uses generated 3D printed models that are available upon request.

### Data and code availability

Representative histological data are presented in this protocol. Additional data available from the lead contact upon request.

## Acknowledgments

This work has been supported by 10.13039/501100000777University of Leeds PhD studentship to B.R.; Yorkshire Bioscience DTP to H.E.S.; 10.13039/100014013UKRI-10.13039/501100000268BBSRC (BB/V016849/1) and 10.13039/100014013UKRI-10.13039/501100000265MRC (MRC/Z50614X/1) to L.K.H.; MRC-Career Development Fellowship (MR/S007288/1) and 10.13039/100010269Wellcome Trust (UNS63234) to B.M.F. and University of Leeds (start-up package) and The 10.13039/501100000288Royal Society (RGS\R2\252055) to P.B.M.d.M. We thank Dr. Cristian Olarte-Sanchez and Dr. Guiseppe D’Agostino for their advice and the staff at the Centre of Biomedical Services, University of Leeds for their assistance.

## Author contributions

Conceptualization, P.B.M.d.M. and B.M.F.; methodology, P.B.M.d.M. and B.M.F.; writing – original draft, P.B.M.d.M.; writing - review and editing, B.R., H.E.S., L.K.H., B.M.F. and P.B.M.d.M.; funding acquisition, P.B.M.d.M., L.K.H., and B.M.F.; resources, P.B.M.d.M. and B.M.F.; supervision and project administration, P.B.M.d.M.

## Declaration of interests

The authors declare no competing interests.
